# Flasks, fibres and flanks – pre-clinical tumour models for predicting clinical antitumour activity

**DOI:** 10.1054/bjoc.2001.1797

**Published:** 2001-05

**Authors:** D R Newell

**Affiliations:** Cancer Research Campaign Cancer Research Unit, Medical School, University of Newcastle, Newcastle, UK


*British Journal of Cancer* (2001) **84**(10), 1289–1290
© 2001 Cancer Research Campaign
doi: 10.1054/ bjoc.2001.1797, available online at http://www.idealibrary.com on IDEAL®

http://www.bjcancer.com

Editorial

### Flasks, fibres and flanks – pre-clinical tumour models for predicting clinical antitumour activity

**DR Newell**

Cancer Research Campaign Cancer Research Unit, Medical School, University of Newcastle, Newcastle, UK

For over 40 years the Developmental Therapeutics Program (DTP) of the National Cancer Institute, USA, has been a global resource for cancer drug developers. Using rodent tumours, then human tumour xenografts and most recently human tumour cell lines, the DTP has developed screening systems with the aim of identifying compounds with potential clinical activity. The tumours and cell lines in the screens were largely selected because of their sensitivity to known, clinically active, anticancer drugs and results from the DTP screens have contributed to the development of many of the cytotoxic drugs in widespread clinical use today. In line with the vast majority of academic drug development groups and pharmaceutical companies, the DTP now focuses on mechanism-based drug discovery, which it is widely anticipated will result in the identification of compounds with greater activity and antitumour specificity than that seen with cytotoxic drugs. Extensive experience with anti-endocrine therapy for the treatment of hormone-dependent tumours, and more recently with trastuzumab and STI571, for the treatment of C-erbB2 over-expressing breast cancer and bcr-abl positive leukaemias, respectively, provides encouragement for target-based drug discovery programmes.

The move away from screening to mechanism-based approaches to drug discovery represents a watershed, and an opportunity to reflect on pre-clinical antitumor models and their utility. The experience of the NCI DTP and Cancer Therapy Evaluation Programme (CTEP) is second to none, and the paper by Johnson and colleagues in this issue has seized this opportunity. In addition to providing a retrospective analysis of the relative abilities of in vitro cell line, hollow fibre and xenograft models to predict clinical activity this paper, as the authors point out, constitutes an important benchmark against which future results will be measured. Whilst we confidently predict that mechanism-based drug development will outperform screening approaches in the discovery of active agents, we need hard data to show that this is the case and the paper from the DTP and CTEP provides these data for pre-clinical studies with conventional cytotoxic drugs. Lastly, even with drugs designed to exploit cancer-related targets, models are still needed to provide confidence that clinical activity can be realistically anticipated, which raises the question of which models to use. With greater experience, it may one day be possible to move in one step from a genomic or proteomic analysis of clinical material through drug design and development to a clinical trial without the use of any pre-clinical tumour models. However, such a day is still some way off and Phase I clinicians, regulatory authorities, ethics committees and patients themselves require reassurance that a new treatment is likely to be of potential benefit.

So, what is the best pre-clinical model? Where should the tumour cells be grown; in tissue culture flasks, intra-peritoneal hollow fibres or implanted in mice? For 39 agents for which Phase II data were available, activity in one-third or more of the xenografts tested was predictive of subsequent Phase II clinical activity. However, with the exception of non-small cell lung cancer, pre-clinical xenograft activity in a particular histological type of cancer was not predictive for the tumour type subsequently found to be sensitive in patients. Thus, on the basis of these data, the clinical Phase II trial cannot be replaced by a pre-clinical study. Although the number of different xenografts per histology was only 7–9, i.e. not the normal Phase II number of 14 tumours, there were nevertheless enough tumours to be confident that pre-clinical studies could not be a substitute for clinical trials, within the limitations of acceptable levels of animal usage.

Given that activity in xenograft models has clinical relevance, the question of what best predicts xenograft activity becomes an issue, in order to minimize the number of animals used in such studies. Overall, 35% of 537 compounds tested had xenograft activity against at least 1 tumour, and for compounds active in 0–3, 4–6, 7–9 or 10 or more intraperitoneal hollow fibres, the level of activity was 28%, 34%, 46% and 63%, respectively. Hence the question becomes whether, or not, improving the likelihood of selecting a xenograft-active compound from 35% to 63% would be worth all the hollow fibre studies? A detailed analysis of numbers of mice used and time required to generate the hollow fibre and xenograft results would help in answering this question, and such an analysis should be undertaken to ensure that the minimum number of experimental animals are being used identify agents for clinical study. An alternative to the hollow fibre model is the use of tumour cell lines grown in vitro, which has the advantage of reducing the use of animals still further, relative to the hollow fibre assay. The authors report the encouraging result that in vitro activity against 6 or more lung or breast cancer cell lines does predict xenograft activity against these tumour types. Furthermore, in vitro tumour type selectivity predicted hollow fibre tumour type selectivity in 5 histologies, and greater in vitro potency was a portent of a greater level of activity against hollow fibres. Lastly, the authors address the issue of whether or not the chemical structure of the compound can be used to predict activity, and show that both MW and hydrogen-bonding capability can influence activity; data which highlights the importance of computational chemistry in drug design and evaluation.

In summary, the DTP and their colleagues in CTEP at the NCI are to be applauded for performing this important analysis. They have set the benchmark against which the pre-clinical aspects of mechanism-based drug development can be compared. For conventional cytotoxic drugs, which constituted the vast majority of the agents studied by the DTP and CTEP, demonstrating activity in xenograft models, which itself can to some extent be predicted by activity in the hollow fibre model and in turn in in vitro cell lines, is of value. Whether this remains the case for the new generation of cancer target-specific drugs remains to be seen and a new experiment, potentially the last, is just beginning.

*© 2001 Cancer Research Campaign*

